# Continuous fabrication of nanostructure arrays for flexible surface enhanced Raman scattering substrate

**DOI:** 10.1038/srep39814

**Published:** 2017-01-04

**Authors:** Chengpeng Zhang, Peiyun Yi, Linfa Peng, Xinmin Lai, Jie Chen, Meizhen Huang, Jun Ni

**Affiliations:** 1 State Key Laboratory of Mechanical System and Vibration, Shanghai Jiao Tong University, Shanghai 200240, P.R. China; 2 Department of Instrument Science and Engineering, Shanghai Jiao Tong University, Shanghai 200240, P.R. China; 3 Department of Mechanical Engineering, University of Michigan, Ann Arbor, MI 48109-2125, USA

## Abstract

Surface-enhanced Raman spectroscopy (SERS) has been a powerful tool for applications including single molecule detection, analytical chemistry, electrochemistry, medical diagnostics and bio-sensing. Especially, flexible SERS substrates are highly desirable for daily-life applications, such as real-time and *in situ* Raman detection of chemical and biological targets, which can be used onto irregular surfaces. However, it is still a major challenge to fabricate the flexible SERS substrate on large-area substrates using a facile and cost-effective technique. The roll-to-roll ultraviolet nanoimprint lithography (R2R UV-NIL) technique provides a solution for the continuous fabrication of flexible SERS substrate due to its high-speed, large-area, high-resolution and high-throughput. In this paper, we presented a facile and cost-effective method to fabricate flexible SERS substrate including the fabrication of polymer nanostructure arrays and the metallization of the polymer nanostructure arrays. The polymer nanostructure arrays were obtained by using R2R UV-NIL technique and anodic aluminum oxide (AAO) mold. The functional SERS substrates were then obtained with Au sputtering on the surface of the polymer nanostructure arrays. The obtained SERS substrates exhibit excellent SERS and flexibility performance. This research can provide a beneficial direction for the continuous production of the flexible SERS substrates.

Surface-enhanced Raman spectroscopy (SERS) has been intensely studied since its discovery in the 1970 s^1^. To date, SERS has been a powerful tool for applications including single molecule detection, analytical chemistry, electrochemistry, medical diagnostics and bio-sensing^2^,^3^,^4^,^5^,^6^. In order to obtain excellent SERS signal enhancement, the uniformity of nanostructures over large areas is quite important since the Raman signal intensity is extremely sensitive to the size, shape, and morphology of the nanostructures on the substrate^7^. Furthermore, reproducibility also largely depends on the uniformity in substrate features which is crucial to sensing in SERS signal enhancement. To address this, numerous techniques have been applied to fabricate uniform nanostructure arrays, for instance, electron beam lithography, interference lithography, focused ion-beam lithography and Anodic Aluminum Oxide (AAO) template deposition^8^,^9^,^10^,^11^,^12^,^13^,^14^,^15^. The gold nanostructure arrays were fabricated on a glass substrate using high-resolution electron-beam lithography and lift-off processes and SERS properties were evaluated of crystal violet molecules on the fabricated structures^9^. The dual interference lithography (IL) was used to fabricate large-area hexagonally ordered ridged nanostructure (HORN) arrays and the metallic HORN arrays exhibited tunable SERS performances with large-scale sample homogeneity^10^. Besides, the large-area ordered arrays of rigid Ag-nanorods (Ag-NRs) can also be obtained on copper base via AAO template-assisted electrochemical deposition and the large-area ordered arrays of Ag-NRs showed excellent SERS performance with uniform electric field enhancement^14^. These techniques can produce uniform nanostructure arrays and in turn offer high and uniform SERS signal enhancements. However, these techniques may show a series of drawbacks such as complicated processing, low throughput and high fabrication costs.

Flexible SERS substrates are highly desirable for daily-life applications^16^, such as real-time and *in situ* Raman detection of chemical and biological targets. Compared with the traditional rigid substrates (e.g. silicon, glass, etc.), flexible substrates, such as plastic or polymers, offer advantages of light weight, transparency, and deformability. Especially, the flexibility enable them can be used onto irregular surfaces. Based on the flexible requirements, there have been some reports on the fabrication of flexible SERS substrates on plastic or polymers. The direct deposition of metal nanoparticles (NPs) on flexible substrates is one method to obtain flexible SERS substrates^17^,^18^, which may have the disadvantages of random distribution and varied density of NPs on the surface resulting in variation in SERS signal enhancement^19^. To obtain high and uniform SERS signal enhancement, the combination of periodic nanostructure arrays and NPs provides one solution and extensive researches have been conducted^16^,^20^,^21^,^22^,^23^,^24^,^25^,^26^,^27^. A two-step block copolymer self-assembly process was demonstrated for fabricating large area SERS-active tapes^16^. Such substrates facilitate the detection and quantification of contaminants on irregular surfaces such as fruit skin, fabrics and other non-planar surfaces. A nanoimprint lithography (NIL) method was reported by sputtering the gold nanoparticles (AuNPs) on the AAO template and then the IPS nanopillars embedded with AuNPs were obtained^21^. The obtained SERS substrate exhibits prominent reproducibility and high sensitivity to Rhodamine 6 G (R6G). Moreover, it possesses excellent transparency and flexibility. The combination of soft lithography and nanosphere lithography was also reported to fabricate flexible and tunable plasmonic nanostructures on polydimethylsiloxane (PDMS)^23^. The nanovoid arrays with Ag layer exhibited a tunable SERS property and an enhancement factor on 10^6^ orders of magnitude was achieved.

Although numerous techniques have been applied to fabricate the SERS substrates, the cost-effective and reproducible fabrication of SERS substrates on a large scale, while maintaining ultrahigh sensitivities, still remains challenging. In this paper, we presented a facile and cost-effective method to fabricate flexible SERS substrate including the fabrication of polymer nanostructure arrays and the metallization of the polymer nanostructure arrays. The polymer nanostructure arrays were obtained by using the AAO mold and roll-to-roll ultraviolet nanoimprint lithography (R2R UV-NIL) technique which has the advantages of high-speed, large-area, high-resolution and high-throughput. The functional SERS substrates were then obtained with Au sputtering on the surface of the polymer nanostructure arrays. The obtained SERS substrates exhibit excellent SERS and flexibility performance. This research can provide a beneficial direction for the continuous production of the flexible SERS substrates.

## Methods

### Materials

The UV-curing resins are generally categorized in two reaction types, radical curing system and cation curing system. In this experiment, the UV-curing resin PHQ53A was used. PHQ53A is a radical curing system resin with fluorinated additive which is produced by Jiangsu Kangde Xin Composite Material Co., Ltd. and it is applied to R2R process, PET substrate and droplet dispensing. As a commonly used flexible substrate, the PET used in the R2R UV-NIL process is 125 μm in thickness and the average transmittance in ultraviolet radiation is above 85%.

### Fabrication of the flexible SERS substrates

**Figure 1:**
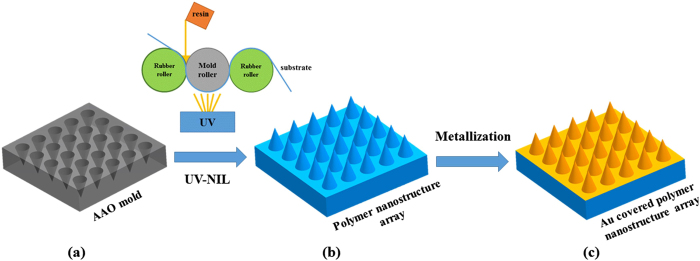
The schematic diagram of fabrication process for the SERS substrates. (**a**) The fabrication of master mold by AAO process, (**b**) the formation of polymer nanostructure array by the UV-NIL process, (**c**) the formation of Au covered polymer nanostructure array by the sputtering process.

The fabrication process of the flexible SERS substrates is illustrated in Fig. 1 with three major phases: (a) the fabrication of the master mold, (b) the formation of polymer nanostructure arrays on a substrate, and (c) finally the metal deposition on polymer nanostructure arrays to generate the flexible SERS substrates. In this experiment, the AAO mold was used as a master mold with diameter 200 nm and depth 350 nm. The AAO process is an inexpensive patterning method for nanofabrication and this provides a solution for the commercialization of flexible SERS substrates. The anodization process was carried out in 2 vol.% phosphoric acid solution at 10 °C under the constant voltage of 120 V for 200 s each time with appropriate magnetic stirring. After anodization, the sample was dipped in 5 vol.% phosphoric acid at 30 °C for 12.5 min to widen the holes. In the present experiment, the five fold alternate repetitions of anodization process and the pore-widening treatment was adopted to form the ordered array of holes with a tapered shape. The five fold repetitions of anodization were carried out under the same conditions and no pore-widening treatment was conducted after the fifth anodization. After that, the AAO mold was thoroughly rinsed with DI water and dried in air.

As a solution for continuous fabrication, R2R UV-NIL technique was used to fabricate polymer nanostructure arrays upon a flexible PET substrate in this research which has the advantages of high-speed, large-area, high-resolution and high-throughput. The plat AAO mold was wrapped around the mold roller in the R2R UV-NIL process. As shown in Fig. 1, the following steps are basically included in the R2R UV-NIL process: the resin is dropped between the mold roller and rubber roller and then is pressed evenly under the effect of the rubber roller pressure. Filling process begins under the effect of the rubber roller pressure and the shape of multi-scale compound eyes is retained with the help of the tension force of PET substrate. The irradiation step starts when the multi-scale compound eyes arrays enters the irradiation range of the UV lamp. The 365 nm UV intensity used in the experiment was about 40 mW/cm^2^. After exposure, the cured polymer nanostructure arrays are released from the mold. Finally, the functional SERS substrates were obtained with Au sputtering on the surface of polymer nanostructure arrays. To further investigate the relation between the thickness of Au coating and the SERS enhancement, Au coating with different thickness was sputtered on the surface of polymer nanostructure arrays. The sputtering durations of 90 s, 180 s, 270 s, and 360 s were adopted, respectively. It is difficult to measure the thickness of Au coating on the surface of the polymer nanostructure arrays and therefore the thickness of Au coating with different sputtering durations was measured on the plat PET substrate in this research. The Au deposition rate was 10 nm/min and the corresponding Au coating thickness was 15, 30, 45, 60 nm, respectively.

### Characterization

The scanning electron microscope (SEM) images were obtained using Zeiss Ultra Plus field emission scanning electron microscope with an electron energy of 5 kV. Atomic force microscope (AFM) images were obtained using a nanoscope scanning probe microscope (Dimension fastscan, Bruker, Germany) under ambient conditions. Au coating was sputtered on the surface of the polymer nanostructure arrays using an ion sputtering apparatus (E-1045, Hitachi, Japan). The light absorption performance of substrates were measured using a spectrophotometer (Shimadzu UV3600, Shimadzu, Japan) combined with an integrating sphere for the 300−800 nm wavelength range. For Raman scattering measurements, 10 μL R6G aqueous solution (10^−6^ M) was dropped onto the substrates and then dried in the dark. Also, the same amount of 10^−1^ M R6G solution was drop-casted on glass to get reference Raman spectra. SERS spectra were collected using a Dispersive Raman Microscope (Senterra R200-L, Bruker Optics, Germany).

## Results

### Characteristics of the nanostructure arrays

**Figure 2:**
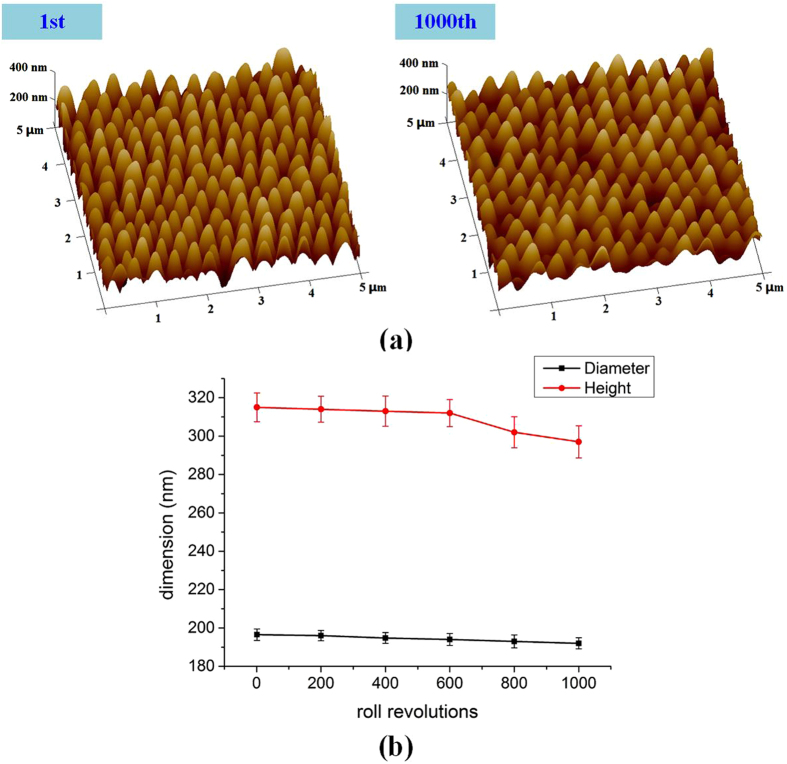
The repeatability investigation of R2R UV-NIL technique to fabricate nanostructure arrays. (**a**) The AFM images of nanostructure arrays at the 1st and 1000th roll revolution, (**b**) the diamters and heights of nanostructure arrays each 200 roll revolutions.

In order to investigate the repeatability of R2R UV-NIL technique to fabricate the nanostructure arrays, we carried out the R2R UV-NIL process up to 1000 roll revolutions (502.5 m). The repetitive experiments were carried out with feeding speed 0.5 m/min, imprinting pressure 6 kg/cm^2^ and mold temperature 25 °C. In this research, the diameters and heights of the nanostructured arrays were measured from AFM images after different roll revolutions, which can obtain structural parameters conveniently, as shown in Fig. 2. The values reported were averaged over at least five measurements performed over different areas of the samples. As shown in Fig. 2(b), the diameters of the nanostructured arrays showed no obvious changes within the 1000 roll revolutions. Moreover, the heights of the nanostructured arrays also showed no obvious changes within the beginning 600 roll revolutions and a slight decline in heights was observed at the 800th and 1000th roll revolution. The slight decline in heights might be attributed to that a small amount of resin was residued in the mold. Although the decline in heights was observed at the 800th and 1000th roll revolution, the changes were not significant and this still indicated that excellent repeatability was accomplished to fabricate nanostructure arrays with R2R UV-NIL process within the 1000 roll revolutions. In order to ensure the consistency of nanostructured arrays as much as possible, the nanostructured arrays obtained within the beginning 10 roll revolutions were chosen to investigate the SERS performance, which were 196 ± 3 nm in diameters and 315 ± 7 nm in heights. According to the experimental results, the structural parameters of the tapered pillars are slightly smaller than that of the nanopores in the AAO mold. The difference in diameter and height might be attributed to the volume shrinkage of the UV-curing resin during the polymerization^28^,^29^.

### SERS performance of the substrate

**Figure 3:**
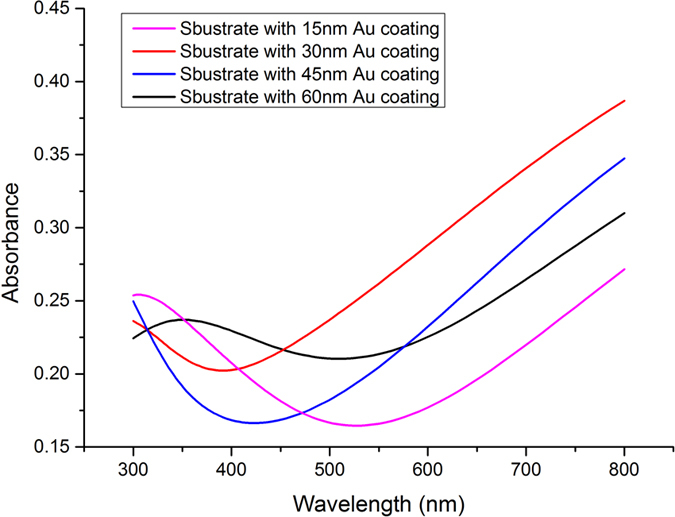
The absorption spectra of substrates with different Au coating thickness within the 300−800 nm wavelength range.

In order to choose the optimal excitation wavelength for Raman spectrum measurement, the light absorption performance of substrates with different Au coating thickness were measured within the 300−800 nm wavelength range, as presented in Fig. 3. As is known, the common laser excitation wavelengths used in the Raman Microscope were 532 nm, 633 nm, and 785 nm^6^,^9^,^14^,^19^, respectively, which was the technical parameters of the instrument. According to the absorption spectra of the substrates, the light absorption at 785 nm wavelength appeared to be higher than that at other wavelengths, which was beneficial to the surface plasmon resonance^30^. Therefore, 1-mW 785-nm excitation was used in this research and the integration time was 10 s for each Raman spectrum. The spectra were collected using a 100x microscope objective (N.A. = 0.9). All the measurements were carried out at room temperature.

**Figure 4:**
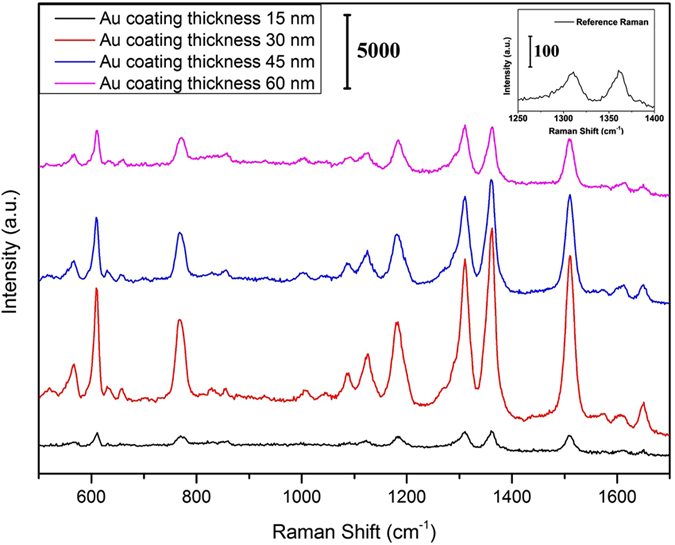
SERS spectra of R6G (10^−6^ M) absorbed on nanostructure arrays with different Au coating thickness. 1-mW 785-nm excitation was used, and the integration time was 10 s for each spectrum.

The SERS performance of the substrate was evaluated by using R6G as the probe molecule in this research. As reported in the previous researches, the SERS enhancement of the substrate is related to the thickness of coating^30^,^31^. To investigate the effect of Au coating thickness on the SERS enhancement, SERS spectra of substrates with different Au coating thickness were measured, as shown in Fig. 4. It can be found that there are six dominant Raman peaks centered at 612, 772, 1185, 1315, 1366 and 1513 cm^−1^ in all the SERS spectra, which are consistent with the Raman signals of R6G molecules^30^,^32^. The Raman bands at 612, 772 and 1185 cm^−1^ can be attributed to the C–C–C ring in-plane vibration mode, the C–H out-of-plane bend mode and the C–H in-plane bending mode of the R6G molecule, respectively. The Raman band at 1315 cm^−1^ can be attributed to the N–H in plane bending mode and the bands at 1366 and 1513 cm^−1^ should correspond to the in-plane C–C stretching modes of R6G^30^,^33^.

**Figure 5:**
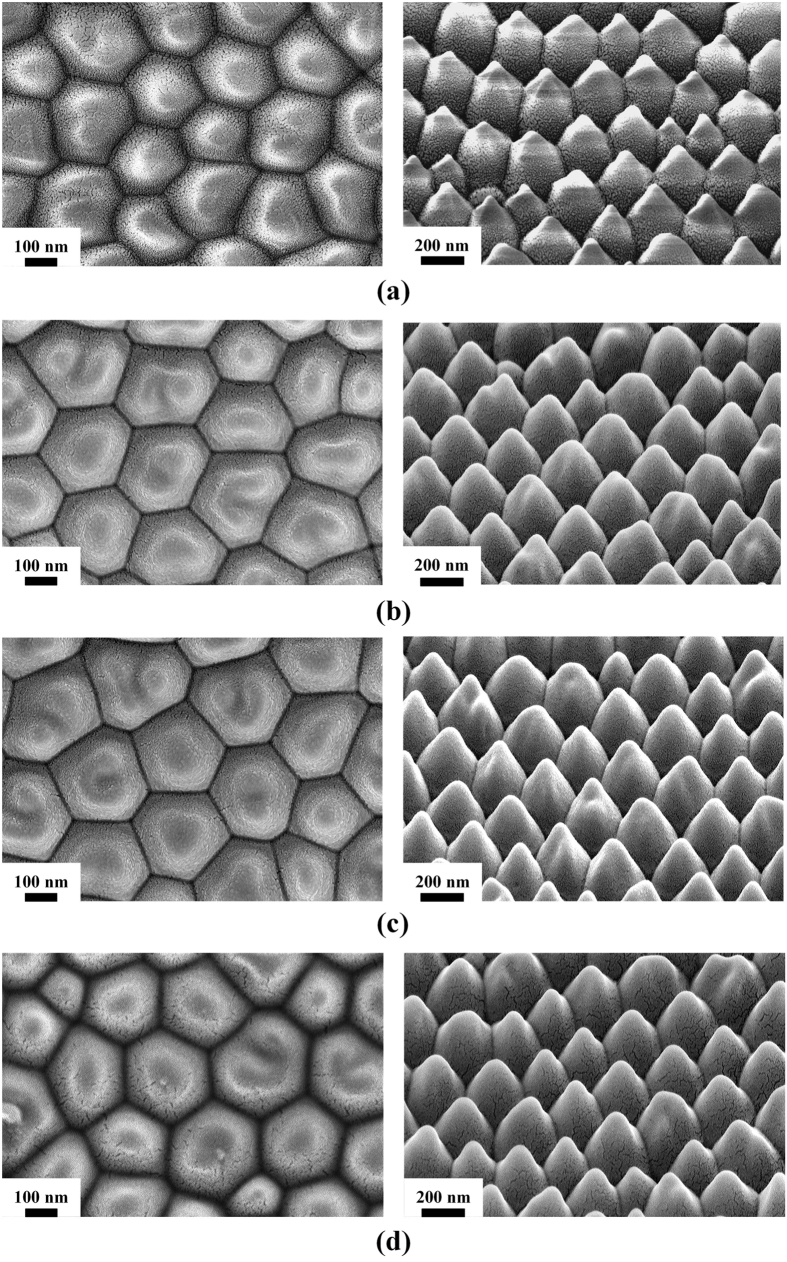
The top view and 45° tilt view SEM images of nanostructure arrays with different Au coating thickness. (**a**) 15 nm, (**b**) 30 nm, (**c**) 45 nm, (**d**) 60 nm.

As shown in Fig. 4, the Raman signal enhancement increases at first and then decreases as the Au coating thickness increases. Among the substrates, the substrate with 30 nm Au coating shows the highest Raman signal enhancement and the substrate with 15 nm Au coating shows the weakest Raman signal enhancement, which might be attributed to the different distribution of Au nanoparticles covered on the polymer nanostructure arrays. Figure 5 showed the SEM images of nanostructure arrays with different Au coating thickness. As shown in Fig. 5, only a small amount of Au nanoparticles were covered on the polymer nanostructure arrays with 15 nm Au coating and this indicated fewer “hot spots” which can cause strong enhancement of the local electric field^34^,^35^. With the increase of Au coating thickness, the Au nanoparticles covered on the polymer nanostructure arrays increase and this make more R6G molecules absorbed on the Au nanoparticles due to larger surface area. At the same time, more Au nanoparticles covered on the polymer nanostructure arrays make the spacing between particles decrease and provide more “hot spots” which can cause strong enhancement of the local electric field^34^,^35^. However, the substrates with 45 nm and 60 nm Au coating also show weaker Raman signal enhancement than the substrate with 30 nm Au coating. This phenomenon might be attributed to that as the Au coating thickness increases, the spacing between particles decrease and even emerge with each other to form the continuous film^36^,^37^, which cause the “hot spots” decrease. Therefore, our experiments indicated that a 30 nm Au coating created an optimal size and separation of Au particles on the polymer nanostructure arrays surface in this research.

To quantitatively evaluate the Raman signal enhancement, we also performed the calculation of the analytical enhancement factor (AEF) for R6G on the substrate according to the following formula: EF = (*I*_SERS_ / *I*_ref_) / (*C*_SERS_ / *C*_ref_)^30^,^38^. *I*_SERS_ and *I*_ref_ correspond to the Raman intensities of R6G on SERS substrate and glass, respectively. *C*_SERS_ and *C*_ref_ denote the molar concentration of R6G in aqueous solution on SERS substrate (10^−6^ M) and glass (10^−1^ M), respectively. In this study, the intensity of the peak at 1366 cm^−1^ was chosen to estimate the EF value. The *I*_SERS_ and *I*_ref_ were obtained directly from the measured Raman spectra. The EF values for substrates with 15 nm, 30 nm, 45 nm, 60 nm Au coating at 1366 cm^−1^ were calculated to be 1.01 × 10^6^, 1.21 × 10^7^, 5.79 × 10^6^, and 2.89 × 10^6^, respectively. Herein, the largest EF value was calculated to be about 1.21 × 10^7^ for substrate with 30 nm Au coating. That might be lower than the EF value reported for R6G molecules on other substrates^39^, where R6G molecules were absorbed on the core–shell nanorods. Many researches have shown that the surface morphology of nanostructures plays an important role in determining the SERS performance and the nanostructures with larger surface area can accommodate more probe molecules^40^,^41^. The tapered pillars obtained in the present research possessed smaller surface area than the nanorods and thus smaller EF value. Although the EF value was a little lower than that of other substrates reported, previous reports have shown that an enhancement factor in the order of 10^7^ to 10^8^ is sufficient for the actual detection application^42^,^43^.

**Figure 6:**
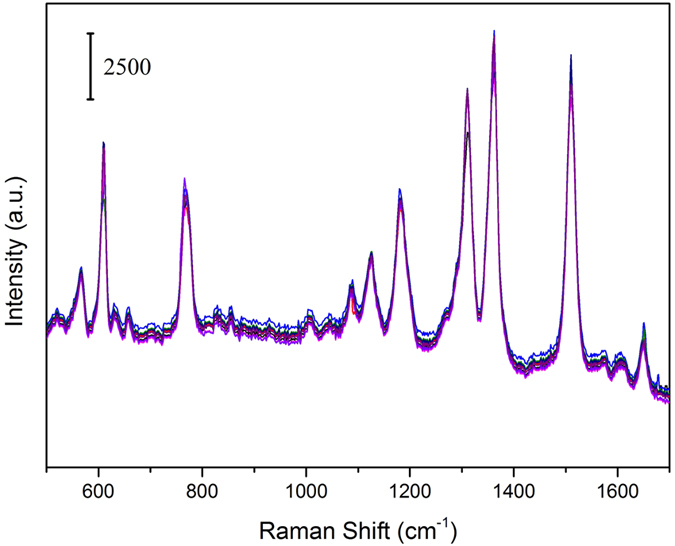
SERS spectra of R6G (10^−6^ M) absorbed on nanostructure arrays collected from 8 randomly selected acquisition points from substrates with 30 nm Au coating. 1-mW 785-nm excitation was used, and the integration time was 10 s for each spectrum.

The reproducibility of Raman signals from the SERS substrate is of great importance for its practical use. To test whether the substrates are able to give reproducible SERS signals of the target molecules, the SERS spectra of R6G molecules with a concentration of 10^−6^ M from 8 randomly selected acquisition points were collected on the substrate with 30 nm Au coating, as shown in Fig. 6. As we can see, the SERS spectra from 8 randomly selected acquisition points show very similar intensities and shapes. Moreover, we calculated the relative standard deviation (RSD) values corresponding to the six major SERS peaks of R6G, as shown in Table 1. For our substrate, all of the RSD values are below 10%. This indicated that the substrate provided excellent reproducibility on the whole substrate surface.

### Flexibility performance of the substrate

**Figure 7:**
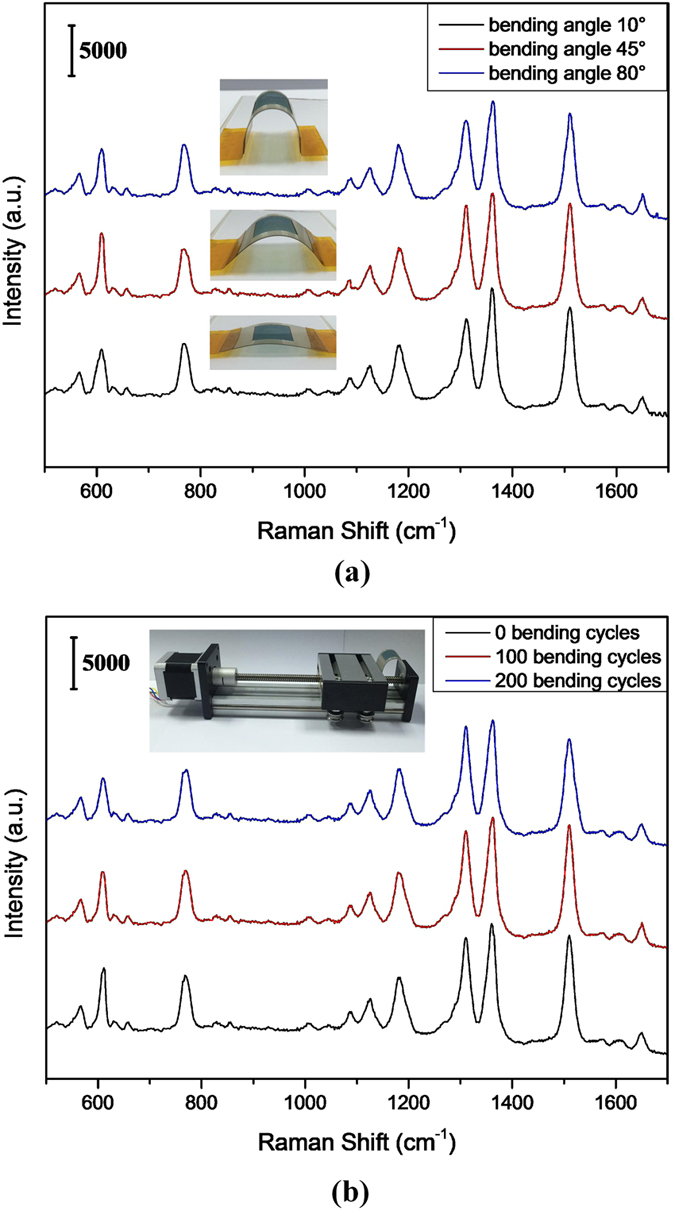
SERS spectra of R6G (10^−6^ M) collected from substrates with 30 nm Au coating. (**a**) With different bending angles, (**b**) with different bending circles and 80° bending angle. 1-mW 785-nm excitation was used, and the integration time was 10 s for each spectrum.

To be used effectively onto irregular surfaces, the flexible substrate should retain superior SERS performance even after mechanical deformation. To investigate the flexibility, the substrate with 30 nm Au coating was adopted and the SERS spectra of R6G (10^−6^ M) obtained from the substrate under different bending angles and different bending cycles were measured. The angle between substrate and horizontal plane is defined as the bending angle. Figure 7(a) show the SERS spectra of R6G (10^−6^ M) obtained from the substrate under different bending angles, which was 10°, 45°, 80°, respectively. As we can see, the SERS spectra under different bending angles show no obvious differences both the SERS signal intensity and peak positions. This phenomenon might be attributed to that the laser spot illuminated on the substrates is very small (about 1 μm) and the area illuminated by the laser is almost planar no matter how large the bending angle is. Figure 7(b) display the SERS spectra of R6G (10^−6^ M) obtained from the substrate under different bending cycles, which were all measured with 80° bending angle. Different bending cycles were accomplished through the screw system, stepping motor, and single-chip microcomputer control. It is obvious that SERS signal intensity and peak positions show no obvious differences even after 200 bending cycles. The above results indicate that the substrate possesses excellent flexibility and stability.

## Conclusions

The R2R UV-NIL technique provides a solution for the continuous fabrication of flexible SERS substrate due to its high-speed, large-area, high-resolution and high-throughput. In this study, the polymer nanostructure arrays were fabricated by using R2R UV-NIL technique and AAO mold. The functional SERS substrates were then obtained with Au sputtering on the surface of the polymer nanostructure arrays. The following conclusions have been determined.

(a) The polymer nanostructure arrays were obtained successfully by using one-step R2R UV-NIL technique and AAO mold. The diameter and height of the tapered pillars used for SERS investigation in this research were 196 ± 3 and 315 ± 7 nm, respectively. The successful fabrication of polymer nanostructure arrays was observed even after 1000 roll revolutions and this indicated excellent repeatability of the R2R UV-NIL process.

(b) Excellent SERS performance and flexibility were achieved with the SERS substrate. In order to obtain optimal SERS enhancement, Au coating with different thickness was sputtered on the surface of polymer nanostructure arrays and finally the substrate with 30 nm Au coating showed the highest SERS enhancement. The largest enhancement factor (EF) for R6G at 1366 cm^−1^ was calculated to be about 1.21 × 10^7^ and the SERS performance showed no obvious differences under different bending angles and different bending cycles.

In this research, the feasibility was verified to fabricate the flexible SERS substrates via one-step R2R imprinting and AAO mold. However, the EF value of the SERS substrate may be lower than that reported for R6G molecules on other substrates. The fabrication of SERS substrate with higher EF value will be further investigated in the following work. This study can provide a beneficial direction for the cost-effective and continuous production of flexible SERS substrates.

## Additional Information

**How to cite this article**: Zhang, C. *et al*. Continuous fabrication of nanostructure arrays for flexible surface enhanced Raman scattering substrate. *Sci. Rep.* **7**, 39814; doi: 10.1038/srep39814 (2017).

**Publisher's note:** Springer Nature remains neutral with regard to jurisdictional claims in published maps and institutional affiliations.

## Figures and Tables

**Table 1:** RSD value for the major peaks of the R6G SERS spectrum.

Peak position (cm^−1^)	612	772	1185	1315	1366	1513
RSD value	9.15%	7.93%	9.52%	8.91%	9.74%	9.45%
